# Effects of spicy food consumption on overweight/obesity, hypertension and blood lipids in China: a meta‐analysis of cross-sectional studies

**DOI:** 10.1186/s12937-023-00857-6

**Published:** 2023-06-08

**Authors:** Mei Wang, Wei Huang, Yong Xu

**Affiliations:** 1 Department of Endocrinology and Metabolism, Taikang Sichuan Hospital, Chengdu, 610000 Sichuan China; 2 Metabolic Vascular Disease Key Laboratory of Sichuan Province, Luzhou, 646000 Sichuan China; 3 Sichuan Clinical Research Center for Nephropathy, Luzhou, 646000 Sichuan China; 4 Department of Endocrinology and Metabolism, Affiliated Hospital of Southwest Medical University, Luzhou, 646000 Sichuan China

**Keywords:** Spicy food, Overweight, Obesity, Hypertension, Blood lipid, Meta-analysis

## Abstract

**Background and objectives:**

Effect of spicy food consumption on health has attracted widespread attention in recent years. However, the relationships between spicy food intake and overweight/obesity, hypertension and blood lipid levels remain unclear. A meta-analysis of available observational studies was conducted in order to explore the associations.

**Methods:**

PubMed, Embase, Cochrane Library and Web of science databases were searched for studies published up to 10 August 2021 without language limitation. The fixed and random effects models were selected to aggregate the effect sizes and 95% confidence intervals (CIs) in this study.

**Results:**

A total of nine observational studies involving 189,817 participants were included. Results from this meta-analysis showed that the highest category of spicy food intake significantly increased the risk of overweight/obesity (pooled Odds Ratio (OR): 1.17; 95% CI: 1.07, 1.28; *P* < 0.001), compared with the lowest category of spicy food intake. Conversely, a remarkable negative association was observed between the highest category of spicy food intake and hypertension (pooled OR: 0.87; 95% CI: 0.81, 0.93; *P* = 0.307). In addition, the highest category of spicy food intake increased the level of low density lipoprotein cholesterol (LDL-C) (weighted mean difference (WMD): 0.21; 95% CI: 0.02, 0.39; *P* = 0.040), and reduced high density lipoprotein cholesterol level (HDL-C) (WMD: -0.06; 95% CI: -0.10, -0.02; *P* = 0.268) concentrations, but it was not related to total cholesterol (TC) (WMD: 0.09; 95% CI: -0.08, 0.26; *P* = 0.071) and triglyceride (TG) (WMD: -0.08; 95% CI: -0.19, 0.02; *P* = 0.333)] levels.

**Conclusion:**

Spicy food intake may have a beneficial effect on hypertension, but adversely affect overweight/obesity, as well as blood lipid levels. However, the results should be interpreted cautiously because the present analyses were based on only observational studies and not intervention studies. More large and high-quality studies in different populations will be needed to verify these associations in the future.

**Supplementary Information:**

The online version contains supplementary material available at 10.1186/s12937-023-00857-6.

## Introduction

With the improvement of health awareness, the relationship between diet and health has received increasing attention in recent times. Particularly, unhealthy diets have been considered to be associated with an increased risk of non-communicable metabolic diseases. For example, higher pro-inflammatory diet(e.g., carbohydrates, proteins, total fat, trans fat, and cholesterol) might be significantly associated with the risk of hypertension [1], type 2 diabetes [2], cardiovascular diseases [3], and metabolic syndrome [4]. Intake of ultra-processed foods significantly might increase the incidence of obesity and all-cause mortality [5, 6]. Additionally, a meta-analysis based on prospective cohort studies indicated that consumption of sugar and artificially sweetened beverages show positive correlation with the incidence of obesity, type 2 diabetes mellitus, hypertension, and all-cause mortality [7]. Therefore, to some extent, reducing the intake of certain foods is essential to prevent the occurrence of diseases.

Spices, such as capsaicin, pepper, Chilli, ginger, garlic, onion, fenugreek, turmeric, are essential parts of food culture around the world, often used as preservatives, colorants, flavor enhancers and pharmaceutical ingredient [8]. Spicy food refers to food with spices for flavoring including chilli sauce, chilli oil, dried capsicum, fresh capsicum and others, which is unique for its pungent flavor, which is characterized as the burning, stinging or tingling sensations elicited by chemical irritants such as capsaicin [9]. In the past decades, numerous studies have been conducted to explore the association between spicy food and their bioactive ingredients and health.

Previous studies have shown that active ingredients such as capsaicin in spicy foods exhibit antioxidant and anti-inflammatory properties by reducing oxidative stress in tissues and organs, reducing vascular permeability and the production of pro-inflammatory cytokines [10–12]. Some studies have shown that consumption of spicy food is associated with reduced risk of cardiovascular disease, ischemic heart disease, cerebrovascular disease, all-cause mortality, diabetes [13–16] and could improve cognitive function in alzheimer's patients [17]. Conversely, a higher level of spicy food intake may increase the risk of various cancers [18], bone fractures [19], and Hyperuricemia [20]. However, the associations between spicy food consumption and overweight/obesity [21–24], hypertension [25–27], and blood lipid levels [28–30] have long been controversial. Hence, it is necessary to fully understand the impact of spicy food consumption on the above diseases.

To our best knowledge, no meta-analysis has been performed on the association between spicy food consumption and overweight/obesity, hypertension, and blood lipid levels. Thus, we conducted a meta-analysis of observational studies, in order to pool available data addressing the association between consumption of spicy food and overweight/obesity, hypertension, and blood lipid levels in general population.

## Methods

This meta-analysis was conducted according to the Preferred Reporting Items for Systematic Reviews and Meta-Analyses (PRISMA) guideline [31] (Supplemental Table 1). Ethical approval is not necessary because this study is a meta-analysis of published studies.

### Search strategy

We systematically searched PubMed, Embase, Cochrane Library and Web of Science to identify eligible articles published until 10 August 2021, without language limitation. The search relevant keywords are as follows: [(Spicy food OR Capsaicin OR Chili OR Chilli OR Pepper) AND (Obesity OR Overweight) OR (Hypertension OR BP OR High blood pressure) OR (Lipid OR Serum lipid OR Blood lipid OR Hyperlipidemia OR Total Cholesterol OR TC OR Low density lipoprotein cholesterol OR LDL-C OR High density lipoprotein cholesterol OR HDL-C OR Triglyceride OR TG)]. Details on our search strategy are presented in Supplemental table 2.

### Inclusion criteria

Studies that were included in this meta-analysis met the following explicit criteria: (1) the study design were cohort, case–control, or cross-sectional studies; (2) consumption of spicy food was considered as the exposure factor; (3) the primary outcomes were overweight/obesity, hypertension, and blood lipid concentrations in the population; (4) studies that provided the relevant effect sizes (ESs) and corresponding 95% confidence intervals (CIs), or mean and standard deviation (SD) of primary outcomes.

### Exclusion criteria

The exclusion criteria are as follows: (1) Duplicate literature, case reports, reviews, editorials, non-population studies; (2) studies that did not consider consumption of spicy food as the exposure factor; (3) studies that did not provide data on the association between consumption of spicy diet and overweight/obesity, hypertension, blood lipid (including total cholesterol(TC), triglyceride(TG), low density lipoprotein cholesterol(LDL-C) and high density lipoprotein cholesterol(HDL-C)); (4) studies in which full test is in unavailable or with incomplete primary data.

### Selection and data extraction

Two investigators searched and screened the literature independently according to the inclusion and exclusion criteria, excluded those that did not meet the inclusion criteria, and cross-checked the extracted information. Any disagreement was resolved by the third party through negotiation.

The following data were extracted from the eligible articles: (1) the corresponding author`s name;(2) year of publication;(3) type of study design; (4) number of participants; (5) sex; (6) the age range of the study population;(7) duration of follow-up;(8) the relevant effect sizes (ESs) and corresponding 95% CIs; (9) the covariates used for adjustment. Nine studies [21–27, 29, 30] included in this meta-analysis reported the associations between the multi-level of spicy food intake and overweight/obesity, hypertension and blood lipid levels. Therefore, we distinguished two levels of spicy food consumption in our study: highest and lowest. The Classifications of intake levels for spicy food followed with the definition in the original studies. The lowest category was defined as the lowest level of spicy food intake (reference group), and in 9 studies, it was defined as “no”, never or few. The highest category was defined as the highest level of spicy food intake, 8 studies [21–25, 27, 29, 30] defined as 6-7 day/week, ≥ 50.1 g/day, ≥ 3times/week, ≥ 5times/week, while 1 study [26] defined as “yes”.

### Assessment of study quality

The methodological quality of observational studies was independently assessed by two investigators, using the Newcastle–Ottawa scale(NOS) [32, 33], which includes selection, comparability and outcome. The results of quality scoring for included articles are in Table 1. Studies with NOS scores ≥ 6 stars are defined as medium to high quality, NOS scores < 6 stars are defined as low quality [34].

**Table 1:** Characteristics of included studies in this meta-analysis

Study ID	Country	Study design	Duration of follow-up (years)	Sample size	Age (mean ± SD or range)	Gender (M/F)	Outcome	Study quality	Variables adjusted or matched
**Li et al** **(2021)**	*China*	Cross-sectional	NR	53,916	52.5 ± 9.9	22,591/31325	hypertension	7	cigarettes consumption, alcohol consumption, physical activity, meat consumption, fruit consumption, BMI, WC, snoring, and sleep duration
**Zhou et al** **(2020)**	China	Cross-sectional	NR	3324	18.51	1372/1952	hypertension	6	age, gender, physical activity, current smoking status, and current alcohol intake
**Na et al** **(2019)**	China	Cross-sectional	NR	57,555	30–79	23,254/34301	overweight and obesity	7	Age, marital status, smoking, alcohol consumption, physical activity, frequency of intake of rice, pasta, cereals, meats, eggs, vegetables, fruits and dairy products
**Wang et al** **(2019)**	China	Cross-sectional	NR	28,773	55.39 ± 12.36	11,721/17052	obesity	8	age, gender, education, marital status, tobacco use, alcohol use, physical activity, and carbohydrate energy intake
**Zhang et al** **(2018)**	China	Cross-sectional	NR	1549	72.7	737/812	blood lipid levels	8	rural residence, ethnicity, age, education, smoking status, alcohol consumption, physical activity, diabetes, BMI, daily dietary intake of daily total energy, daily vegetable intake, and daily fruit intake
**Xue et al** **(2018)**	China	Cross-sectional	NR	9273	18–99	4423/4850	hypertension	7	age, gender, nationality, education, BMI, smoking status, alcohol consumption, physical activity, dietary intake of total energy, vegetable, fruits, cereals, and meats
**Page et al** **(2017)**	China	Cohort	9	12,970	≥ 20	6485/6485	obesity	8	intake of fat, smoking, alcohol drinking, income, urban, education, physical activity, and dietary patterns (traditional south pattern; a modern dietary pattern)
**Wang et al** **(2017)**	China	Cross-sectional	NR	15,683	56.32 ± 9.66	5907/9776	obesity	8	age, education, tobacco use, alcohol use, physical activity, marital status, energy intake, various spices, high fat intake, and more vegetables and fruits intake
Zhu et al (2017)	China	Cross-sectional	NR	6774	18–65	3184/3590	blood lipid levels	7	age, sex, nationality, education, smoking status, alcohol consumption, BMI, physical activity, dietary intake of total energy, vegetables, dietary intakes of carbohydrate, fat, and cholesterol

*NR* Not reported

### Statistical analysis

In this meta-analysis, we identified hazard ratios (HRs) as equivalent to odds ratios (ORs) [35]. In the spicy food intake and blood lipid analysis, the effect index was the weighted mean difference (WMD) and corresponding 95% CIs. The heterogeneity between studies was assessed by using the Q test and I^2^ statistics [36], and I^2^ values 25%, 50%, 75% respectively represent zero, moderate and high heterogeneity. If the statistical heterogeneity was significant among studies, the random effect model was selected to estimate the ESs and its 95% CIs. Otherwise, the fixed effect model was selected. All statistical analyses were conducted with Stata software (version 12.0). P < 0.05 was considered the level of statistical significance.

## Results

### Literature search and study characteristics

**Fig. 1:**
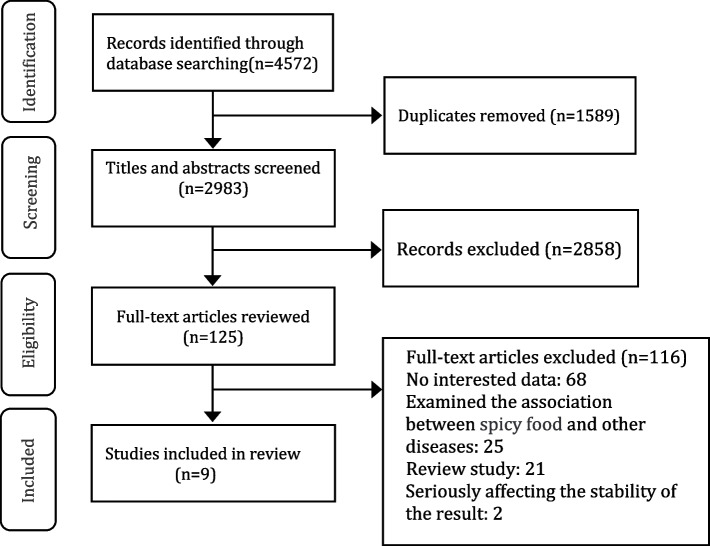
Flow diagram for the selection of eligible observational articles

We initially identified 4572 studies in PubMed (*n* = 1082), Cochrane Library (*n* = 275), Embase (*n* = 1424) and Web of science (*n* = 1791) databases, and no studies were found through other sources. Of these, 1589 duplicate articles were excluded, and 2858 articles were excluded after reviewing the title and abstract. Among these remaining articles that underwent full text review, 68 articles did not provide interested data, 25 articles examined the association between spicy food and other diseases, 21 articles were reviews, 2 article was found to have a significant effect on the stability of the results in sensitivity analysis. Finally, 9 articles [21–27, 29, 30] meeting the inclusion and exclusion criteria were included in this meta-analysis. The process of literature screening and results are shown in the flow diagram (Fig. 1). The characteristics of the included studies are shown in Table 1. All of 9 studies were observational studies, including 2cohort studies, 7 cross-sectional studies. All of the studies included both men and women.

### The effect of spicy food consumption on overweight/obesity

**Fig. 2:**
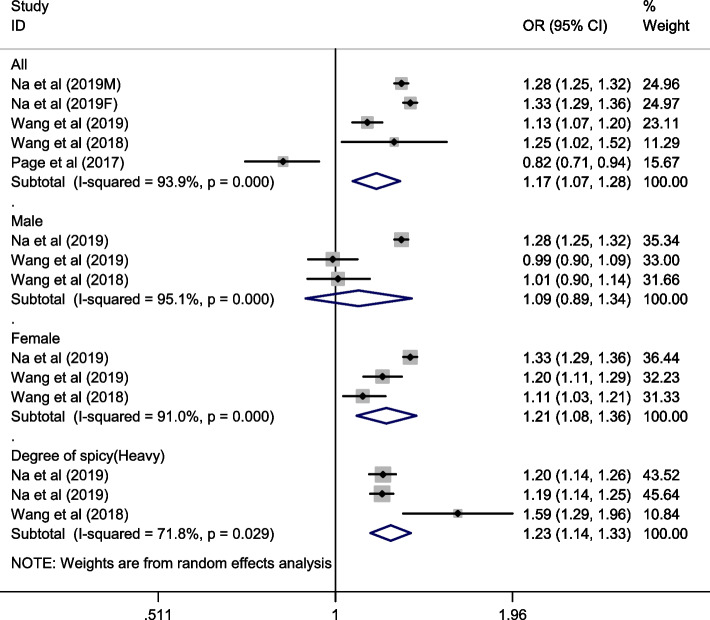
Forest plot of the association between consumption and flavour of spicy food and overweight and obesity

A total of four studies explored the relationship between spicy food consumption and overweight or obesity. As shown in Fig. 2, data from the meta-analysis showed that the highest category of spicy food intake was associated with an increased risk of overweight/obesity (pooled OR: 1.17; 95% CI: 1.07, 1.28; I^2^ = 93.9%, *P* < 0.001). All studies except one reported that spicy food consumption was positively associated with overweight/obesity. Subgroup analyses showed that the association between the highest category of spicy food intake and overweight/obesity was significant for females (pooled OR 1.21; 95% CI: 1.08–1.36; I^2^ = 91.0%, *P* < 0.001), but not for males (pooled OR 1.09; 95% CI: 0.89, 1.34; I^2^ = 95.1%, *P* < 0.001). Further analyses suggested that compared with no spicy flavor, heavy spicy flavor of spicy food significantly increased the risk of overweight/obesity (pooled OR: 1.23; 95% CI:1.14, 1.33; I^2^ = 71.8%, *P* = 0.029).

### The effect of spicy food consumption on hypertension

**Fig. 3:**
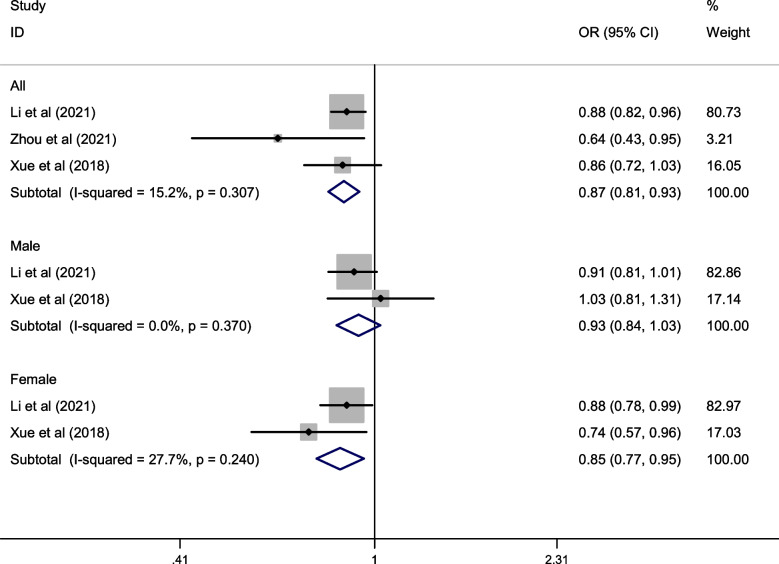
Forest plot of the association between spicy food consumption and hypertension

Three studies assessed spicy food consumption and hypertension incidence. As shown in Fig. 3, the highest category of spicy food intake significantly decreased the risk of hypertension (pooled OR: 0.87; 95% CI: 0.81, 0.93; I^2^ = 15.2%, *P* = 0.307), with all studies except that of Wang et al. [27] reporting a positive association between spicy food consumption and hypertension. Subgroup analyses showed that the highest category of spicy food intake was associated with a significant reduction in the risk of hypertension in females (pooled OR: 0.85; 95% CI: 0.77, 0.95; I^2^ = 27.7%, *P* = 0.240), but was not related to males (pooled OR: 0.93; 95% CI: 0.84, 1.03; I^2^ = 0.0%, *P* = 0.370). No evidence of statistically significant heterogeneity was found among studies (I^2^ = 15.2%, *P*-heterogeneity = 0.307).

### The effect of spicy food consumption on blood lipid concentrations

**Fig. 4:**
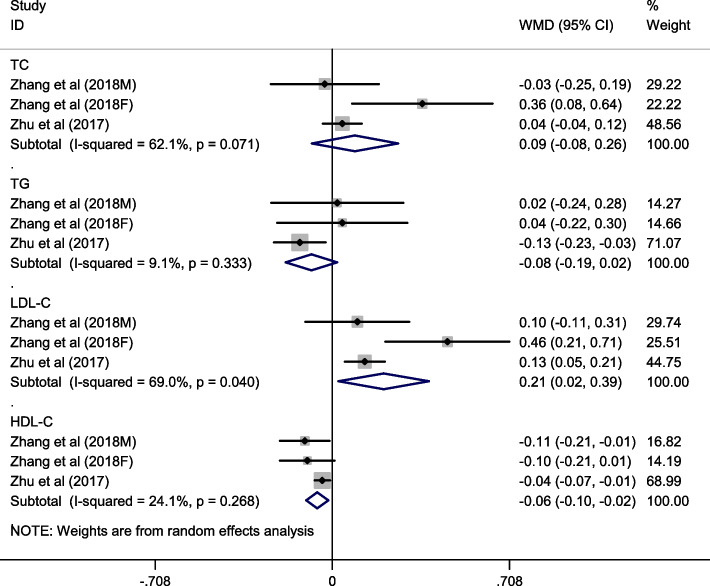
Forest plot of the association between spicy food consumption and blood lipid level (including total cholesterol (TC), triglyceride (TG), low density lipoprotein cholesterol (LDL-C) and high density lipoprotein cholesterol (HDL-C))

Two studies with 3 effect sizes examined the association between spicy food consumption and blood lipid levels. As shown in Fig. 4, the highest category of spicy food intake increased LDL-C level(SMD: 0.21; 95% CI: 0.02, 0.39; I^2^ = 69%, *P* = 0.040). On the contrary, regular or high consumption of spicy food reduced HDL-C level (WMD: -0.06; 95% CI: -0.10, -0.02; I^2^ = 24.1%, *P* = 0.268). Nevertheless, no significant reduction was observed in TC (WMD: 0.09; 95% CI: -0.08, 0.26; I^2^ = 62.1%, *P* = 0.071) and TG concentrations (WMD: -0.08; 95% CI: -0.19, 0.02; I^2^ = 9.1%, *P* = 0.333).

### Sensitivity analysis and publication bias

The summary effects for overweight/ obesity and hypertension were not influenced by any particular study. Nevertheless, sensitivity analyses showed that the study of Wang et al. [28] significantly affected the robustness of the meta-analysis results (TC: from *P* = 0.000, I^2^ = 83.3% to *P* = 0.071, I^2^ = 62.1%; LDL-C: from *P* = 0.003, I^2^ = 78.4% to *P* = 0.040, I^2^ = 69%; HDL-C: from *P* = 0.004, I^2^ = 77.8% to *P* = 0.268, I^2^ = 24.1%). Thus, it was excluded from this meta-analysis. Results from Begg's rank correlation test suggested that no evidence of publication bias were detected in the meta‐analysis of overweight/obesity, hypertension, and blood lipids (TC, TG, LDL‐C, HDL‐C) (*p* > 0.05).

## Discussion

To the best of our knowledge, this is the first meta-analysis to systematically evaluate the association between spicy food consumption and overweight/obesity, hypertension and blood lipid levels. In this meta-analysis, we observed that the highest category of spicy food intake was significantly associated with increased risk of overweight/obesity. In addition, it was observed that spicy food consumption was negatively correlated with hypertension. For the effect on blood lipids, spicy food consumption significantly increased LDL-C concentration, slightly lowered HDL-C concentration; however, no remarkable effect was detected on TC and TG concentrations.

Previous studies have demonstrated that spicy food consumption has potential benefits for weight management in short-term intervention studies with a small sample size from Western countries [37–40], including: (1) increased lipid oxidation and inhibit adipogenesis; (2) activated brown adipose tissue activity and induced thermogenesis; (3) suppressed appetite and increased satiety regulated by neuronal circuits in the hypothalamus; (4) modulating the function of gastrointestinal tract and gut microbiome [41, 42]. However, data on the impact of spicy food consumption on overweight/obesity in Asian populations are scarce. A population-based epidemiologic study involving half a million Chinese people found that the covariates-adjusted BMI, percentage body fat (BF%), waist circumference (WC), and WC/height ratio (WHtR) significantly increased with increasing frequency, strength, and duration of spicy food eating in males and females [43]. In contrast, one study conducted in healthy overweight female subjects found that there were no significant changes in body weight and body mass index in the capsaicin intervention group compared with placebo [44]. Clinical trials have also shown no significant differences in the indicators of obesity between the placebo and capsaicin groups [44, 45]. This meta-analysis of evidence from observational studies observed that regular or high spicy food consumption was positively correlated with overweight/obesity, compared with no or less spicy food intake, especially in females. One of the reasons for the contradiction in results could be that the majority of the experiments with a small sample size used isolated supplemental or research grade capsaicinoids for experimental interventions, while observational studies selected natural food sources as the interventions.

Several potential mechanisms can be used to describe the association between spicy food consumption and the risk of overweight/obesity. Spicy foods are more likely to be meat and less likely to be vegetables, and the consumption of spicy foods may increase meat intake, thereby increasing the risk of overweight/obesity [46]. Additionally, spicy food intake can increase sweet cravings, which leads to significant weight gain [39]. Moreover, chili oil is used for seasoning in Chinese cuisine, and the intake of chili oil can be accompanied by an increase in the intake of carbohydrate-rich foods to relieve the burning sensation, which also might lead to weight gain [24, 43]. Whereas, the above are just speculations, and the exact underlying mechanism between consumption of spicy food and overweight/obesity is unclear. Further study is expected to explore the mechanism.

Evidence from early animal studies and clinical studies suggested that spicy food consumption might enhance energy expenditure and fat oxidation [40, 45, 47, 48]. On the contrary, other studies have shown no significant effect of spicy food consumption on energy expenditure or fat oxidation [49, 50]. One study included in the present meta-analysis observed that individuals with chilli consumption below 20 g/d and above 50 g/d had reduced and increased energy intake respectively compared with non-consumers [22]. The potential theory to explain this phenomenon may be that at low level of capsaicin intake there is sensitization of vagal afferents leading to increased satiety signaling and reduced food intake. Further, above a certain threshold capsaicin desensitises vagal afferents leading to reduced satiety signaling and an increase in food intake. It seems to suggest that the amount of spicy food intake might have a significant effect on the health outcomes. Furthermore, multiple human studies have demonstrated that spicy food has beneficial effect on energy balance by varying the intake of fat, carbohydrate and protein. Wang et al. [23] analyzed the role of energy intake in mediating the association between spicy food intake frequency and the risk of abdominal obesity. The result revealed that fat energy intake partially mediated the relationship between spicy food intake frequency and abdominal obesity, while there were no significant effects mediated by protein energy intake, carbohydrate energy intake and total energy intake.

Association between spicy food consumption and other health outcomes has also been examined in human population. As was shown in the present study, spicy food consumption had beneficial impact on hypertension. Our findings are consistent with previous studies. Capsaicin could reduce the levels of blood pressure in the models of high-salt-induced hypertension mice and renovascular hypertension [51, 52]. Similar result was found in hypertensive volunteers [53]. Interestingly, our subgroup analysis showed that spicy food consumption reduced the incidence of hypertension in females, but not in males. Fried food consumption significantly increased the risk of hypertension in women, while no significant association was found in men [54]. The study conducted in Korea found that a diet rich in whole grains and legumes is inversely associated with the risk of hypertension in women but not in men [55]. It might be suggested that blood pressure in females might be more easily influenced by diet, when compared to males. The potential explanation of the relationship between spicy food and hypertension might be that transient receptor potential vanilloid 1(TRPV1) activation by spicy food could increase urinary sodium excretion [51], reduce vascular lipid accumulation and attenuate atherosclerosis [56], and improve endothelium-dependent vasorelaxation to prevent hypertension [57], meanwhile spicy food could decrease individual salt preferences and daily salt intake [58].

The effect of spicy food on blood lipid has also received extensive attention. Evidence of the relationship between spicy food consumption and blood lipid levels was summarized in this meta-analysis. The results showed that spicy food intake respectively increased and reduced LDL-C and HDL-C concentrations, while it had no significant effect on TC and TG levels. Low HDL-C may be associated with low intake of fat and cholesterol and high intake of carbohydrate in the diet of Chinese adults [59]. A cross-sectional study, excluded from the present meta-analysis due to significant impact on the heterogeneity, indicated that spicy food intake was mildly associated with increased risk of abnormal TAG level, but significantly associated with decreased risk of abnormal TC and non-HDL levels [28]. One mechanism underlying the cholesterol-lowering activity of capsaicinoids is that they can stimulate faecal excretion of bile acids. Additionally, two studies examined the associations between different degrees of spicy flavour and serum lipid profiles [28, 30]. Zhu et al. have observed that the degree of pungency was positively associated with serum TG but showed no relationship with serum TC [30]. Wang et al. [28] have also demonstrated that spicy flavour was consistently associated with decreased TC and non-HDL-cholesterol levels but mildly associated with elevated TG levels. It might be suggested that spicy food flavour and intake have varying degrees of influence on blood lipid levels. Although some studies have explored the relationship between spicy foods and lipids, the mechanism is still not fully established. Further study is expected to explore the mechanism.

Spicy-food intake has been shown to affect various human physiological systems and diseases. For example, results of a meta-analysis of prospective cohort studies show that the consumption of spicy food is associated with a 12% and 18% reduction in all-cause mortality and death from cardiac diseases, respectively [60]. Besides, a case–control study conducted in India proved that spicy foods intake is a risk factor for nonalcoholic fatty liver disease [61]. Another study has suggested that high consumption of spicy foods is associated with a greater risk of uninvestigated heartburn in men, but not in women [62]. Additionally, spicy stimulation has an analgesia effect on adults that persists even after the taste stimulation stops, whereas a long-term spicy diet can reduce the human basal pain threshold [63]. Therefore, more attention should be paid to the impact of diet on health, especially spicy food.

### Strengths and limitations

Although this meta-analysis represents the most comprehensive summary and analysis of the available evidence regarding the relationship between spicy food intake and the risk of overweight/obesity, hypertension and blood lipid levels, several potential limitations need to be considered. First of all, because this meta-analysis is based on data from observational studies, some unmeasured residual confounding factors may have affected the conclusions of this study. Secondly, the participants of all included studies in this meta-analysis are Chinese, so there may be racial differences. Thirdly, fewer studies were included for each index, which may result in insufficient sample size. Finally, spicy food consumption of all included studies is assessed through Food Frequency Questionnaire, 24 h dietary recall or a standardized questionnaire, which may have potential recall biases and limitation in assessing culture-specific foods.

## Conclusion

Pooled data from a small number of published observational studies suggest that spicy food consumption is associated with increased risk of overweight/obesity, but with reduced risk of hypertension. Meanwhile, spicy food consumption significantly increases and lower LDL-C and HDL-C concentrations, respectively; while there is no remarkable difference in TC and TG concentrations. Although many effects have been found in relation to spicy food consumption in adults, more large and high-quality studies are essential in order to confirm these results.

## Supplementary Information

**Additional file 1: Supplemental table 1.** PRISMA checklist. **Supplemental table 2.** Search strategy to identify observational studies reporting the associations of spicy food intake and overweight/obesity, hypertension and blood lipid levels.

## Data Availability

All the data in this meta-analysis are from published studies and we take responsibilities for the data integration process and the accuracy of the statistical analyses process.
