# Supplementary material for: Effects of spicy food consumption on overweight/obesity, hypertension and blood lipids in China: a meta‐analysis of cross-sectional studies
Source: Nutr J. 2023 Jun 8;22:29. doi: 10.1186/s12937-023-00857-6 (PMC10249255; doi:10.1186/s12937-023-00857-6)
Supplement: Supplementary file 1 — Additional file 1: Supplemental table 1. PRISMA checklist. Supplemental table 2. Search strategy to identify observational studies reporting the associations of spicy food intake and overweight/obesity, hypertension and blood lipid levels. [file 12937_2023_857_MOESM1_ESM.docx]

**Supplemental Materials**

**Supplemental table 1. PRISMA checklist**

| **Section/topic** | **Item No** | **Checklist item** | **Reported on page No** |
| --- | --- | --- | --- |
| Title | 1 | Identify the report as a systematic review, meta-analysis, or both | Title |
| Structured summary | 2 | Provide a structured summary including, as applicable, background, objectives, data sources, study eligibility criteria, participants, interventions, study appraisal, synthesis methods, results, limitations, conclusions and implications of key findings, and systematic review registration number | Introduction |
| Rationale | 3 | Describe the rationale for the review in the context of what is already known | Introduction |
| Objectives | 4 | Provide an explicit statement of questions being addressed with reference to participants, interventions, comparisons, outcomes, and study design (PICOS) | Introduction |
| Protocol and registration | 5 | Indicate if a review protocol exists, if and where it can be accessed (e.  g, Web address), and, if available, provide registration information including registration number | Not applicable |
| Eligibility  criteria | 6 | Specify study characteristics (such as PICOS and length of follow-up) and report characteristics (such as years considered, language, and publication status) used as criteria for eligibility, giving rationale | Methods |
| Information sources | 7 | Describe all information sources (such as databases with dates of coverage and contact with study authors to identify additional studies) in the search and date last searched | Methods |
| Search | 8 | Present full electronic search strategy for at least one database, including any limits used, such that it could be repeated | Methods |
| Study selection | 9 | State the process for selecting studies (that is, screening, eligibility, included in systematic review, and, if applicable, included in the meta-analysis) | Methods |
| Data collection process | 10 | Describe method of data extraction from reports (such as piloted forms, independently, in duplicate) and any processes for obtaining and confirming data from investigators | Methods |
| Data items | 11 | List and define all variables for which data were sought (such as PICOS and funding sources) and any assumptions and simplifications made | Methods |
| Risk of bias in individual studies | 12 | Describe methods used for assessing risk of bias of individual studies (including specification of whether this was done at the study or outcome level), and how this information is to be used in any data synthesis | Methods |
| Summary measures | 13 | State the principal summary measures (such as risk ratio and difference in means) | Methods |
| Synthesis of results | 14 | Describe the methods of handling data and combining results of studies, if done, including measures of consistency (such as I^2^ statistic) for each meta-analysis | Methods |
| Risk of bias across studies | 15 | Specify any assessment of risk of bias that may affect the cumulative evidence (such as publication bias and selective reporting within studies) | Methods |
| Additional analyses | 16 | Describe methods of additional analyses (such as sensitivity or subgroup analyses and meta-regression), if done, indicating which were pre-specified | Results |
| Study selection | 17 | Give numbers of studies screened, assessed for eligibility, and included in the review, with reasons for exclusions at each stage, ideally with a flow diagram | Results and Figure |
| Study characteristics | 18 | For each study, the present characteristics for which data were extracted (such as study size, PICOS, and follow-up period) and provide the citations | Table |
| Risk of bias within studies | 19 | Present data on risk of bias of each study and, if available, any outcome-level assessment (see item 12) | Results |
| Results of individual studies | 20 | For all outcomes considered (benefits or harms), present for each study (a) simple summary data for each intervention group and (b) effect estimates and confidence intervals, ideally with a forest plot | Figure |
| Synthesis of results | 21 | Present results of each meta-analysis done, including confidence intervals and measures of consistency | Figure |
| Risk of bias across studies | 22 | Present results of any assessment of risk of bias across studies (see item 15) | Results |
| Additional analysis | 23 | Give results of additional analyses, if done (such as sensitivity or subgroup analyses, meta-regression) (see item 16) | Results |
| Summary of evidence | 24 | Summarize the main findings including the strength of evidence for each main outcome; consider their relevance to key groups (such as health care providers, users, and policy makers) | Discussion |
| Limitations | 25 | Discuss limitations at study and outcome level (such as risk of bias), and at review level (such as incomplete retrieval of identified research and reporting bias) | Discussion |
| Conclusions | 26 | Provide a general interpretation of the results in the context of other evidence and implications for future research | Discussion |
| Funding | 27 | Describe sources of funding for the systematic review and other support (such as supply of data) and role of funders for the systematic review | Discussion |

**Supplemental table 2.** Search strategy to identify observational studies reporting the associations of spicy food intake and overweight and obesity, hypertension and blood lipid levels

| Database | Search terms |
| --- | --- |
| PubMed | (Capsaicin[MeSH] OR Spicy food[Title/Abstract] OR Chili[Title/Abstract] OR Chilli[Title/Abstract] OR Pepper[Tile/Abstract]) AND ((Obesity[MeSH] OR Overweight[MeSH]) OR (Hypertension[MeSH] OR BP[Title/Abstract] OR High blood pressure[Title/Abstract]) OR (Lipid[MeSH] OR Cholesterol[MeSH] OR Hyperlipidemia[MeSH] OR Serum lipid[Title/Abstract] OR Blood lipid[Title/Abstract] OR Total Cholesterol[Title/Abstract] OR TC[Title/Abstract] OR Low-density lipoprotein cholesterol[Title/Abstract] OR LDL-C [Title/Abstract] OR High-density lipoprotein cholesterol[Title/Abstract] OR HDL-C[Title/Abstract] Triglyceride[Title/Abstract] OR TG[Title/Abstract] )) |
| Cochrane Library | 1 (Spicy food):ti,ab,kw OR (Capsaicin):ti,ab,kw OR (Chili):ti,ab,kw OR (Chilli):ti,ab,kw OR (Pepper):ti,ab,kw  2 (Obesity):ti,ab,kw OR (Overweight):ti,ab,kw  3 (Hypertension):ti,ab,kw OR (High blood pressure):ti,ab,kw  4 (Lipid):ti,ab,kw OR (Serum lipid):ti,ab,kw OR (Blood lipid):ti,ab,kw OR (Hyperlipidemia):ti,ab,kw OR (Cholesterol):ti,ab,kw OR (Total Cholesterol):ti,ab,kw OR (TC):ti,ab,kw OR (Low-density lipoprotein cholesterol):ti,ab,kw OR (LDL-C):ti,ab,kw OR (High-density lipoprotein cholesterol):ti,ab,kw OR (HDL-C):ti,ab,kw OR(Triglyceride):ti,ab,kw OR (TG):ti,ab,kw  5 2 OR 3 OR 4  6 1 AND 5 |
| EMBASE  Web of Science | 1 exp Spicy food/  2 (Spicy food OR Capsaicin OR Chili OR Chilli OR Pepper).ab,ti  3 1 OR 2  4 exp Obesity/  5 exp Overweight/  6 4 OR 5  7 exp Hypertension/  8 (Hypertension or High blood pressure).ab,ti  9 7 OR 8  10 exp Lipid/  11 (Lipid OR Serum lipid OR Blood lipid OR Hyperlipidemia OR Cholesterol OR Total Cholesterol OR TC OR Low-density lipoprotein cholesterol OR LDL-C OR  High-density lipoprotein cholesterol OR HDL-C OR Triglyceride OR TG).ab,ti  12 10 OR 11  13 6 OR 9 OR 12  14 3 AND 13  1 TS=(Spicy food) OR TS=(Capsaicin) OR TS=(Chili) OR TS=(Chilli) OR TS=(Pepper)  2 TS=(Obesity) OR TS=(Overweight)  3 TS=(Hypertension)  4 TS=(High blood pressure) OR TS=(Lipid) OR TS=(Serum lipid) OR TS=(Blood lipid) OR TS=(Hyperlipidemia) OR TS=(Cholesterol) OR TS=(Total Cholesterol) OR TS=(TC) OR TS=(Low-density lipoprotein cholesterol) OR TS=(LDL-C) OR TS=(High-density lipoprotein cholesterol) OR TS=(HDL-C) OR TS=(Triglyceride) OR TS=(TG)  5 2 OR 3 OR 4  6 1 AND 5 |
